# A Case of Multiple Acquired Smooth Muscle Hamartoma With Depressed Appearance

**DOI:** 10.7759/cureus.90622

**Published:** 2025-08-20

**Authors:** Kaoru Chiba, Itaru Dekio, Isami Uno, Yoshimasa Nobeyama, Akihiko Asahina

**Affiliations:** 1 Dermatology, The Jikei University School of Medicine, Tokyo, JPN

**Keywords:** acquired cutaneous tumor, depressed macules, dermatopathology, multiple pigmented lesions, smooth muscle hamartoma

## Abstract

Smooth muscle hamartoma (SMH) is a benign cutaneous tumor that typically presents at birth as a solitary lesion in the lumbosacral region. Acquired cases with multiple lesions and subtle depression are exceedingly rare. We report a 40-year-old male with a seven-year history of multiple pigmented macules with slight depression on the trunk and extremities. Histopathology of a back lesion revealed irregularly distributed bundles of pale eosinophilic smooth muscle fibers in the upper dermis, positive for α-smooth muscle actin (α-SMA). Basal hyperpigmentation was observed, without swelling of collagen fibers or atrophic eccrine glands. Differential diagnoses such as Becker's nevus and atrophoderma of Pasini and Pierini were excluded based on clinical and histological findings. Taken together, the patient was diagnosed with SMH. Although excision may be considered for solitary lesions, it was not performed in our patient due to multiple lesions. No progression was observed over two years of follow-up. This case illustrates a unique acquired SMH with multiple lesions and depressed appearance, emphasizing the importance of recognizing this entity in the differential diagnosis of multiple pigmented macules in adults.

## Introduction

Smooth muscle hamartoma (SMH) is a benign cutaneous tumor characterized by the proliferation of smooth muscle bundles, first reported by Sourreil et al. in 1969 [1]. Most cases are congenital and solitary, presenting at birth or in early infancy as pale brown macules or papules on the lumbosacral region [2]. Clinically, SMH typically shows slightly indistinct borders, with hypertrichosis and pseudo-Darier sign frequently observed in congenital cases [3]. Histologically, interlacing smooth muscle bundles are found in the reticular dermis, as evidenced by positive staining for α-smooth muscle actin (α-SMA) [4]. Main differential diagnoses include morphea, Becker’s nevus, and atrophoderma of Pasini-Pierini. To the best of our knowledge, only 26 acquired cases have been reported [3], and only 11 cases with multiple lesions have been described in the literature. In this report, we present a unique case of acquired SMH with multiple lesions and a depressed appearance.

This article was previously posted to the Authorea preprint server on September 1, 2023.

## Case presentation

**Figure 1:**
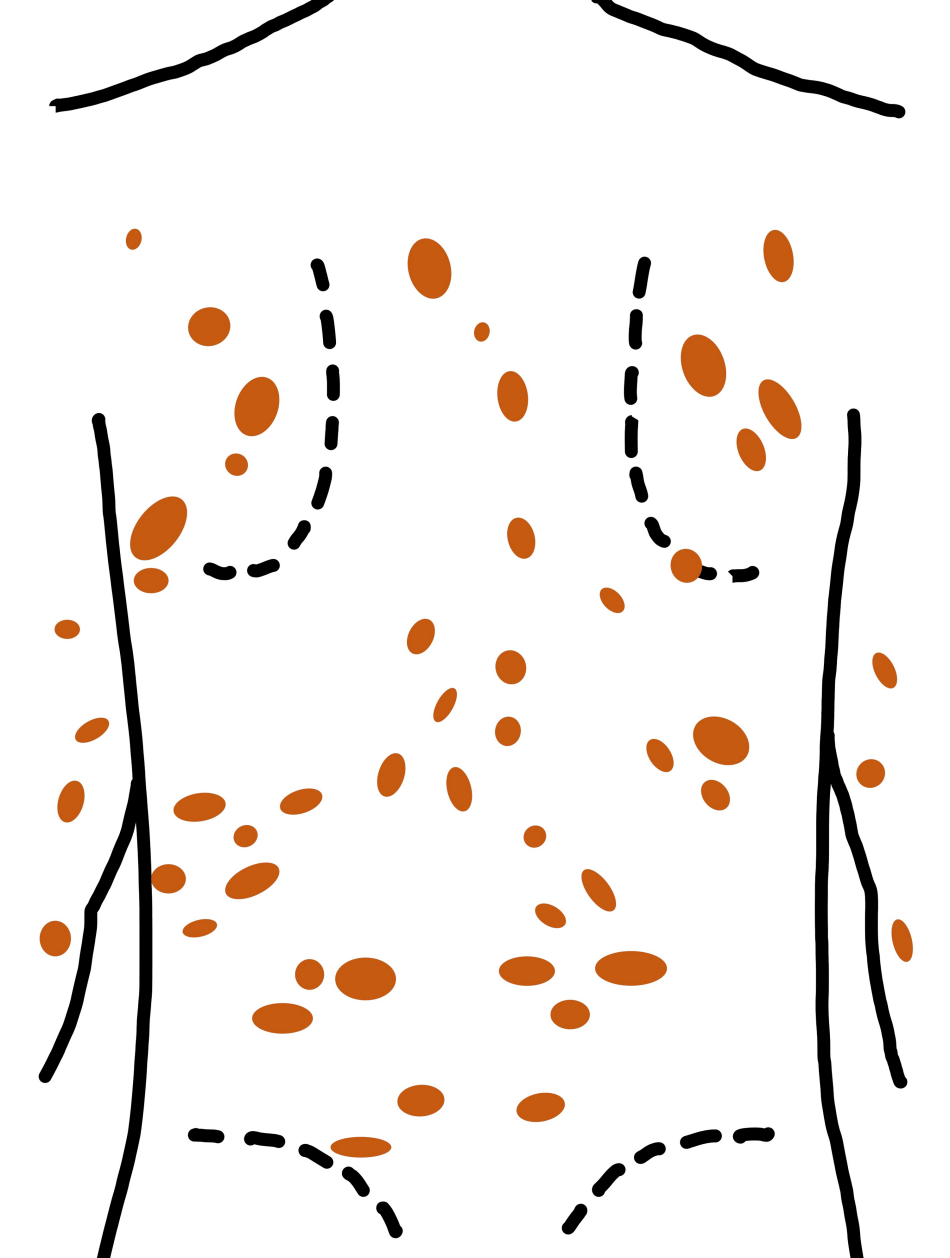
Distribution of the macules (schematic illustration by the authors shown in place of a clinical photograph, as consent was obtained only for partial views). Multiple pigmented macules, ranging in diameter from 1 to 3 cm, were observed on the back.

**Figure 2:**
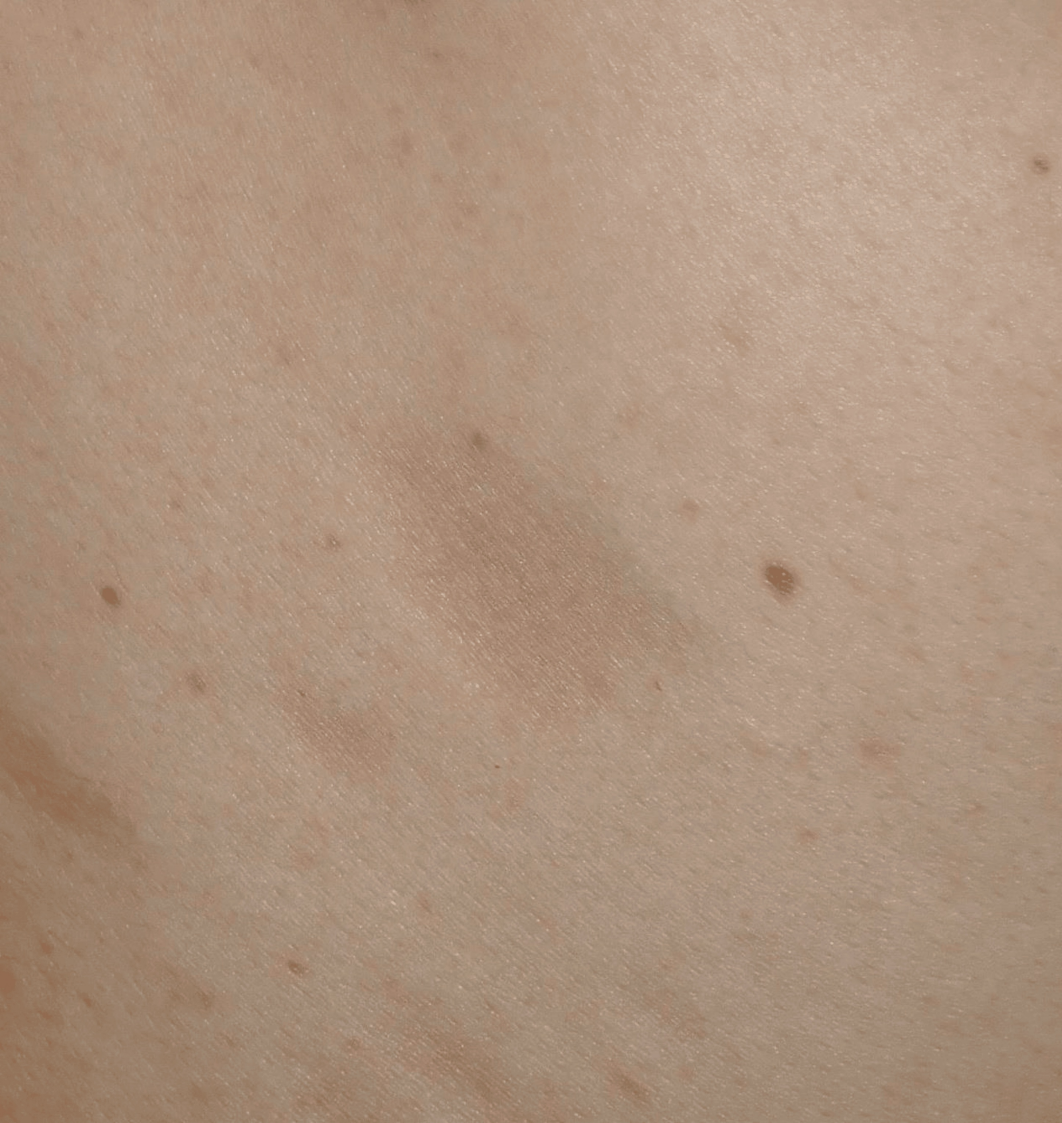
Appearance of a macule on the right upper back. The macules exhibited slight depression.

A 40-year-old French man presented with a seven-year history of pigmented macules on the trunk and extremities. He had previously been diagnosed with morphea elsewhere. There was no history of injections to the lesions or similar findings in his family. Physical examination revealed multiple 1-3 cm slightly depressed, oval-shaped pigmented macules with slightly indistinct borders, lacking hypertrichosis, on the trunk and extremities (Figures 1, 2).

**Figure 3:**
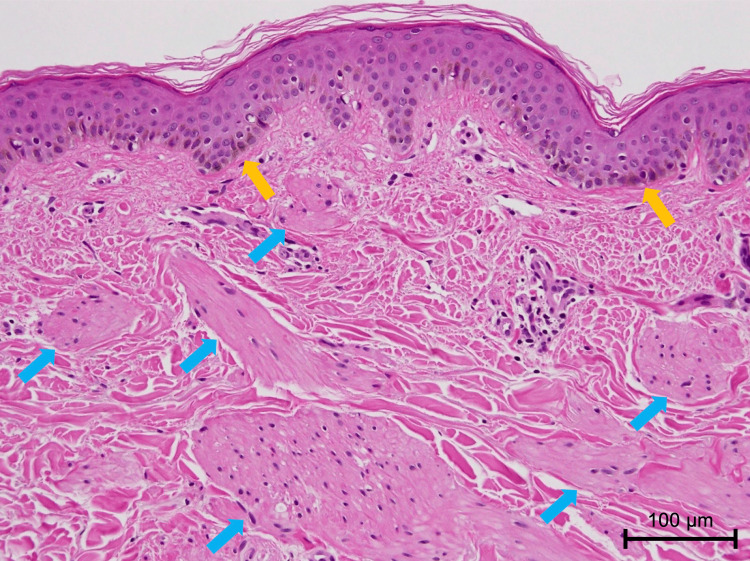
Histopathological findings on hematoxylin and eosin staining (×100). The upper dermis contained irregularly distributed, variably sized bundles of pale eosinophilic structures with oval-shaped nuclei (blue arrows), along with basal hyperpigmentation (yellow arrows).

Laboratory findings were normal, except for a slight increase in thymus and activation-regulated chemokine (TARC) levels, and antinuclear antibody was negative. Histopathology of a biopsy from a macule on the back showed irregularly distributed, variably sized bundles of pale eosinophilic structures with oval nuclei in the upper dermis (Figure 3). Serial sections revealed that these structures were present independently of hair follicle structures. No epidermal changes were observed. Basal hyperpigmentation was present, but there was no swelling of collagen fibers or atrophic eccrine glands trapped within collagen fibers.

**Figure 4:**
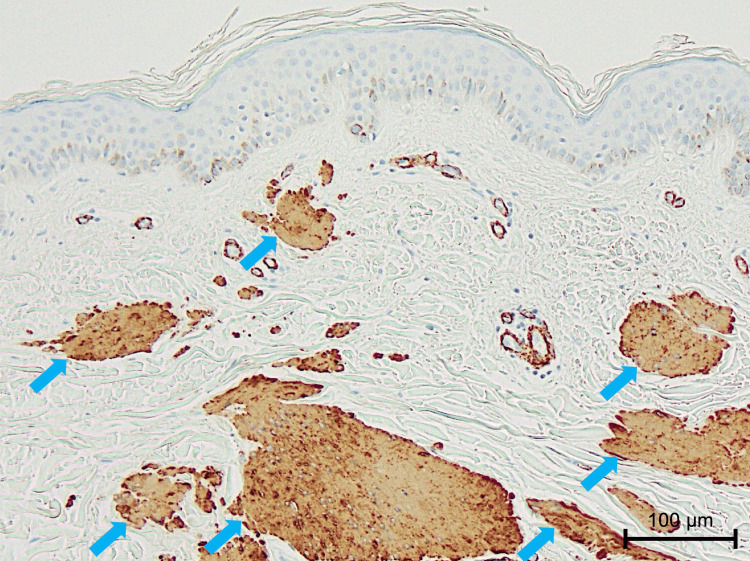
Histopathological findings on immunohistochemistry for α-smooth muscle actin (×100). The bundled structures exhibited positive staining (blue arrows), suggestive of smooth muscle proliferation.

Masson trichrome staining showed these bundles were less stained than surrounding dermal collagen fibers, and they were positive for α-smooth muscle actin (Figure 4).

These findings suggested aberrant proliferation of mature smooth muscle in the dermis, leading to a diagnosis of SMH. A second-site biopsy was considered to confirm the diagnosis, but was not performed at the patient’s request. The patient was followed for two years without apparent changes in cutaneous manifestations.

## Discussion

This case exhibited an atypical clinical presentation of SMH, distinguished by three key features: multiple lesions, acquired onset, and depressed appearance. The presence of multiple lesions initially suggested multiple Becker's nevi as a differential diagnosis [5]. However, the absence of hypertrichosis and epidermal changes was inconsistent with Becker’s nevi. Although a few cases of multiple SMH have been reported in localized areas such as the head [6], widespread involvement remains exceedingly rare. While multiple SMH have also been described in a single family [7,8], there was no evidence of familial occurrence in our case.

The onset at 33 years of age is particularly notable, given that SMH is typically congenital. Reports of acquired cases remain limited. A 2022 review identified 26 cases of acquired SMH worldwide, with 14 of these occurring after the age of 30 [3].

The depressed appearance of the lesions necessitated a thorough differential diagnosis. Atrophoderma of Pasini and Pierini was ruled out based on the presence of significant smooth muscle proliferation [9]. Furthermore, lilac rings indicative of scleroderma en plaques, subcutaneous indurations associated with lupus profundus, or erythema with hyper- or hypo-keratosis suggestive of discoid lupus erythematosus were all absent. These findings supported the diagnosis of SMH. Notably, reports of SMH with depressed appearance, as observed in our case, are extremely limited [10].

A plausible hypothesis for the multifocal, adult-onset presentation is somatic mosaicism [11], in which a postzygotic mutation in a smooth muscle regulatory pathway leads to a mixture of normal and mutated dermal cells. These mutant cells may have been widely distributed but remained clinically inapparent for many years. Later in life, factors such as physical pressure or mild inflammation might have triggered the growth of smooth muscle cells, resulting in visible lesions. Moreover, the slight depression of each lesion may result from the contraction of proliferated smooth muscle bundles exerting traction on the overlying skin.

Acquired SMH requires no treatment, as no cases of malignant transformation have been reported to date. Although excision may be considered for solitary lesions for cosmetic reasons [3], this approach was not feasible in our patient due to the widespread distribution of lesions.

A limitation of this report is that smooth muscle proliferation was assessed only with α-SMA staining. Because α-SMA alone cannot exclude myofibroblastic differentiation, additional markers such as desmin and h-caldesmon could have provided stronger confirmation of smooth muscle lineage.

## Conclusions

In conclusion, we reported a unique case of acquired SMH presenting with multiple lesions and a depressed appearance. Given that SMH is generally congenital and solitary, this case was atypical in terms of onset and distribution. The diagnosis was primarily based on characteristic histopathological features, supported by the absence of signs indicative of other atrophic or connective tissue diseases. We acknowledge that α-SMA alone does not exclude myofibroblastic processes, and additional markers such as desmin and h-caldesmon could have better confirmed smooth muscle lineage. A possible hypothesis for this presentation is somatic mosaicism, with a postzygotic mutation leading to latent dermal smooth muscle proliferation, later activated by physical or inflammatory triggers. Dermatologists should include SMH in the differential diagnosis of multiple depressed pigmented macules in adults.
